# Supplementary material for: Guiding antibiotics towards their target using bacteriophage proteins
Source: Nat Commun. 2024 Jun 20;15:5287. doi: 10.1038/s41467-024-49603-4 (PMC11190222; doi:10.1038/s41467-024-49603-4)
Supplement: Supplementary file 1 — Supplementary information [file 41467_2024_49603_MOESM1_ESM.pdf]

Supplementary information

Guiding antibiotics towards their target using bacteriophage proteins

Xinghong Zhao[1,2,6,*], Xinyi Zhong[1,2,6], Shinong Yang[1,2,6], Jiarong Deng[1,2], Kai Deng[1,2], Zhengqun Huang[1,2], Yuanfeng Li[3], Zhongqiong Yin[1,*], Yong Liu [4,*], Jakob H. Viel[5], Hongping Wan[1,2,*]

[1] Center for Sustainable Antimicrobials, Department of Pharmacy, College of Veterinary Medicine, Sichuan Agricultural University, Chengdu, 611130, China.

[2] Center for Infectious Diseases Control (CIDC), College of Veterinary Medicine, Sichuan Agricultural University, Chengdu, 611130, China.

[3] Translational Medicine Laboratory, The First Affiliated Hospital of Wenzhou Medical University, Wenzhou, Zhejiang, 325035, China.

[4] Wenzhou Institute, University of Chinese Academy of Sciences, Wenzhou, Zhejiang, 325001 China.

[5] Groningen Biomolecular Sciences and Biotechnology Institute, University of Groningen, 9747AG Groningen, Netherlands.

[6] These authors contributed equally to this work.

* Correspondence: xinghong.zhao@sicau.edu.cn (X. Zhao); yinzhongq@163.com (Z. Yin); y.liu@ucas.ac.cn (Y. Liu); hpwan@sicau.edu.cn (H. Wan)

Methods

Reagents

ClonExpress Ultra One Step Cloning Kit (Cat No. C115-02), 2 × Phanta Max Master Mix (Dye Plus) (Cat No. P525-02), DL5000 DNA Marker (Cat No. MD102-02), DL15000 DNA Marker (Cat No. MD103-01), FastPure Gel DNA Extraction Mini Kit (Cat No. DC301-01), FastPure Plasmid Mini Kit (Cat No. DC201-01) were purchased from Vazyme Biotech Co., Ltd. (Nanjing, China); Luria–Bertani broth (LB, Cat No. HB0128) and Tryptic Soy Broth (TSB, Cat No. HB4114) were purchased from Qingdao Hope Bio-Technology CO., Ltd.; Prestained Protein Marker (10-180kDa) (Cat No. BL712A), 3,3′,5,5′-Tetramethylbenzidine (TMB) Liquid Substrate System (Cat No. BL728A), and 4',6-diamidino-2-phenylindole (DAPI, Cat No. BS097-10mg) were purchased from Labgic Bioechnology Co., Ltd. (Beijing, China); Isopropyl-β-D-thiogalactopyranoside (IPTG) (Cat No. I8070), Ni-NTA agarose HP (Cat No. S9320), ColorMixed Protein Marker(11-245KDa) (Cat No. PR1920), CCK-8 Cell Proliferation and Cytotoxicity Assay Kit (Cat No. CA1210), Bicinchoninic Acid Protein Assay Kit (Cat No. PC0020) and ECL Western Blotting Substrate (Cat No. PE0010) were purchased from Beijing Solarbio & Technology Co., Ltd. (Beijing, China); His-Tag (6*His) Monoclonal antibody (Cat No. 66005-1-lg), horseradish peroxidase-conjugated Goat Anti-Mouse IgG (Cat No. SA00001-1), horseradish peroxidase-conjugated Goat Anti-Mouse IgM (Cat No. SA00012-6), and horseradish peroxidase-conjugated Goat Anti-Mouse IgA (Cat No. SA00012-7) were purchased from Proteintech Group, Inc (Rosemont, USA); cetyltrimethylammonium bromide (CTAB, Cat No. Cat no. 57-09-0) was purchased

from Shanghai Macklin Biochemical Co., Ltd. (Shanghai China); tetraethyl orthosilicate (TEOS**,** Cat No. 78-10-4) and Poly(ethylene glycol) (N-hydroxysuccinimide 5-pentanoate) ether N′-(3-maleimidopropionyl)aminoethane (MW=5000Da, MAL-PEG_{5000}-NHS, Cat No. 757853-100MG) were purchased from Sigma-Aldrich (St. Louis, USA); N-hydroxysuccinimide containing red fluorescent dye DyLight 633 was purchased form ThermoFisher scientific (Cat No. 46414, St. Louis, USA); (3-aminopropyl)triethoxysilane (APTES, Cat No. A107147-100ml), 1,2-distearoyl-sn-glycero-3-phosphoethanolamine-N-[amino(polyethylene glycol)-2000] (MW=2000Da, DSPE-PEG_{2000}, Cat No. D163634-500mg), 1,2-distearoyl-sn-glycero-3-phosphoethanolamine-N-[maleimide(polyethylene glycol)-2000] (MW=2000Da, DSPE-PEG_{2000}-MAL, Cat No. D163619-500mg), imipenem (Cat No. I304258-1g), ampicillin (Cat No. A105483-25g), and rifampicin (Cat No. R105455-25g) were purchased from Shanghai Aladdin Biochemical Techology Co., Ltd. (Shanghai China); Soya Phosphatidyl Choline was purchased from Lipoid GmbH (Reg. Certificate No. F20090050, Ludwigshafen, Germany); Cholesterol (Cat No. A610122-0050) was purchased from Shenggong Bioengineering Co., Ltd. (Shanghai, China); Tribromoethanol (Cat No. M2940) was purchased from Nanjing AIBI Bio-Technology Co., Ltd. (Nanjing, China).

Preparation of antibiotic-loaded nanodelivery systems

Preparation of UPSNs and UPSN-NH_2

Urchin-like porous silica nanoparticles (UPSNs) were synthesized according to a previous study with minor modifications [1]. Briefly, the template

cetyltrimethylammonium bromide (900 mg) and sodium salicylate (300 mg) were dissolved in 60 mL ultrapure water and vigorously stirred at 80°C. Subsequently, 150 mg triethylamine (TEA) was added to the above solution and kept stirring until the TEA was thoroughly dissolved. Then, 6mL tetraethyl orthosilicate (TEOS) was added to the above mixture as the silica source. After stirring for 9 h, the products were collected by centrifugation at 12000 g for 15 min and washed 3 times with ultrapure water to remove the residual agents. The template in UPSNs was removed by calcination at 600 °C for 8 h, and the final product was stored in a sealed and dry container for further use. In order to prepare UPSN-NH_2 [2], 100 mg of UPSNs was dispersed in 10 mL of anhydrous toluene, and 1 mL (3-aminopropyl)triethoxysilane (APTES) was added. The mixture was then refluxed at 100 °C under a nitrogen atmosphere for 24 hours. The resulting product (UPSN-NH_2) was collected through centrifugation at 12000 g for 15 min, washed 3 times with ethanol, and dried under vacuum.

Preparation rifampicin-loaded UPSNs

UPSN-NH_2 (10 mg) was added to an aqueous solution of rifampicin (20mL, 0.5mg/mL), and incubated at room temperature for 24 h with rotation at 60 rpm. After that, the excess reagent was removed by centrifugation at 8,000 g for 10 min, yielding Rif@UPSN.

Coupling of gCBD$_{SA97}$ to Rif@UPSN

Rif@UPSN was resuspended in 1 mL of 0.1 M phosphate buffer at pH 7.4 to a final concentration of 10 mg/mL. After that, 0.2 mg of MAL-PEG$_{5000}$-NHS was added to the suspension and incubated at room temperature for 1 h with stirring, to generate MAL-

PEG_{5000} conjugated Rif@UPSN. After removing the excess reagent by centrifuging at 8,000 g for 10 min, the maleimide-activated UPSNs were resuspended in 1 mL of 0.1 M phosphate buffer at pH 7.4. Subsequently, the suspension was mixed with 200 µL of $gCBD_{SA97}$ at a concentration of 8 mg/mL and stirred for 2 h at room temperature to conjugate $gCBD_{SA97}$ via designed free cysteine residue at the N-terminus of this protein, to generate Rif@UPSN@CBD_{SA97}. After removing the excess proteins by centrifugation at 8,000 g for 10 min, the engineered Rif@UPSN@CBD_{SA97} was washed with PBS for three times. Then, the Rif@UPSN@CBD_{SA97} was lyophilized and stored at -20 °C before use. Sterilized sodium chloride solution or phosphate-buffered saline solution is recommended to prepare the nanomedicine resuspensions for administration.

Liposomal coating

Liposomes were prepared by the thin-film hydration method. Briefly, soya phosphatidyl choline, cholesterol, DSPE-PEG_{2000}, and DSPE-PEG_{2000}-MAL were dissolved in trichloromethane with a molar ratio of 54.5: 30: 5: 0.5. The concentration of soya phosphatidyl choline was 10 mg/mL. Subsequently, the mixture was transferred to a round bottom flask, and the organic solvent was removed by rotary evaporation. During evaporation, the lipid membrane was evenly distributed on the flask wall. After that, the lipid membrane was hydrated with Rif@UPSN suspension, 10 mL at a concentration of 1 mg/mL. After sonication for 5 min under a power of 400 W with 2s on and 2s off, maleimide-activated Rif@LUN was obtained by centrifugation.

Conjugation of $gRBP_{P545}$ to maleimide-activated Rif@LUN

Rif@LUN@RBP_{P545} was engineered by mixing 200 µL of $gRBP_{P545}$ (at a concentration of 15 mg/mL) with 1 mL of maleimide-activated Rif@LUN (at a concentration of 10 mg/mL) and incubation at room temperature for 2h with rotation during which $gRBP_{P545}$ was conjugated to maleimide-activated Rif@LUN via the designed free cysteine residue at the N-terminus of this protein. The reaction buffer was 0.1 M phosphate buffer at pH 7.4. After removing the excess proteins by centrifugation at 8,000 g for 10 min, the engineered Rif@LUN@RBP_{P545} was washed with PBS for three times. Then, the Rif@LUN@RBP_{P545} was lyophilized and stored at -20 °C before use. Sterilized sodium chloride solution or phosphate-buffered saline solution is recommended to prepare the nanomedicine resuspensions for administration.

Characterization of antibiotic-loaded nanodelivery systems

Drug loading capacity and in *vitro* release profile

The rifampicin loading content was measured by dissolving 2 mg of the rifampicin-loaded nanoparticles in 1 mL acetonitrile, followed by the addition of 4 mL methanol to dissolve rifampicin. After sonication in an ultrasound bath at 150 W for 5 min, the mixture was centrifuged at 12 000 g for 10 min. The rifampicin content was calculated by measuring the concentration of supernatant samples using high performance liquid chromatography (HPLC). The loading capacity (LC) was calculated according to the following equations: LC% = (Total content of encapsulated rifampicin / total content of nanoparticles)*100%.

The release profiles of rifampicin from rifampicin-loaded nanoparticles were monitored over a duration of 72 h. Briefly, 5 mg of Rif@UPSN, Rif@UPSN@CBD_{SA97}, Rif@LUN, or Rif@LUN@RBP_{P545} was dispersed uniformly in 5 mL of PBS at 37 ℃ with shaking at 60 rpm. Following a desired time point, 2 mL of the supernatant was removed from the release buffer after centrifugation and replaced by 2 mL of fresh PBS. The amount of rifampicin released from the nanoparticles was analyzed by HPLC, and the drug release profile was drawn accordingly.

The concentration of rifampicin solution samples was measured using an Agilent 1260 Infinity HPLC system with a Phenomenex Aeris™ C18 column (250 × 4.6 mm, 3.6 µm particle size, 100 Å pore size). The mobile phase was a mixture of citrate buffer (0.075 M KH_2PO_4 and 1 M citric acid), methanol, and acetonitrile with a ratio of 40:30:30 (v/v/v), and the used flow rate was 1 mL per min for separation over 15 min. The wavelength of the UV detector was adjusted to 254 nm to monitor the change in rifampicin concentration, and the column temperature was maintained at 30 °C during the analysis.

Bicinchoninic acid (BCA) assay

The quantity of $gCBD_{SA97}$ and $gRBP_{P545}$ on the nano vehicles was verified by a BCA assay. The bicinchoninic acid assay was performed according to the manufacturer's guidelines (Cat No. PC0020, Beijing Solarbio & Technology Co., Ltd., Beijing, China). Briefly, the standard protein bovine serum albumin (BSA) was diluted with PBS at a series of concentrations of 2000, 1500, 1000, 750, 500, 250, 125, and 25 µg/mL. After treating the samples (20 µL) with BCA working solution (200 µL) in a 96-well plate for

30 min at 37 °C, the absorbance values were measured using a Thermo Scientific Varioskan Flash multimode microplate reader at a wavelength of 562 nm. The amount of $gCBD_{SA97}$ and $gRBP_{P545}$ on the nano vehicles was calculated using the BSA standard protein as a reference.

Biosafety assessment

Mammalian cytotoxicity

The cytotoxicity of the two distinct nanodelivery systems, $LUN@RBPP_{545}$ and $UPSN@CBD_{SA97}$, was evaluated on the cell viability of two cell lines, hepatoblastoma cell line (Hep G2, ATCC HB-8065) and human embryonic kidney 293T (HEK-293T, ATCC CRL-3216), by using CCK-8 Cell Proliferation and Cytotoxicity Assay Kit (CA1210, Solarbio, China). HepG2 and HEK-293T cells [in Dulbecco's Modified Eagle's medium (DMEM) supplemented with 10% Fetal Bovine Serum] were seeded into 96-well plates and incubated at 37 °C with 5% CO_2. After 24 h, the medium was replaced with fresh medium (DMEM with 2% FBS, 100 µL per well) containing different concentrations of $LUN@RBPP_{545}$ or $UPSN@CBD_{SA97}$. After 24 h incubation, the CCK-8 reagent was added to the cultures according to the manufacturer's instructions, and the plates were incubated at 37 °C for 2 h with 5% CO_2. Subsequently, the absorbance values were measured using a Thermo Scientific Varioskan Flash multimode microplate reader at a wavelength of 450nm.

Hemolytic activity assay

Hemolytic activity assay was performed using the method described in a previous study [3]. In short, erythrocytes were freshly isolated from the blood of healthy New

Zealand white rabbits (9–10 months old, 3.0–3.5 kg) and washed with 0.1 M PBS three times. Subsequently, LUN@RBP$_{P545}$ or UPSN@CBD$_{SA97}$ were added at final concentrations of 512, 128, 64, 32, 16, and 0 μg/mL in PBS containing 2% (v/v) erythrocytes. The cells were incubated at 37 °C for 1 h and centrifuged for 15 min at 3,000 g. The supernatant was transferred to a 96-well plate, and the absorbance was measured at a wavelength of 570 nm using a Thermo Scientific Varioskan Flash multimode microplate reader. The absorbance relative to the positive control, which was treated with 10% Triton X-100, was defined as the percentage of hemolysis. Representative examples from three replicates are shown.

In *vivo* safety studies

Thirty healthy SPF-grade ICR mice (female, 6 weeks, 20±2 g) were randomly divided into three groups (n = 10) and treated with LUN@RBP$_{P545}$ (20 mg/kg), UPSN@CBD$_{SA97}$ (20 mg/kg), and sterile saline, respectively. The body weights of mice were recorded daily for consecutive 7 d. After 7 d of observation, the mice were euthanized, and serum and whole blood were collected for the comprehensive metabolic panel and blood cell counts. In addition, the hearts, livers, spleens, lungs, and kidneys were harvested and fixed in 4% paraformaldehyde, sectioned into 5 μm, and stained with hematoxylin and eosin (H&E) for histological analysis.

Reapplication potential study of the engineered nanodelivery systems

LUN@RBPP$_{545}$ reapplication potential study

Thirty healthy SPF-grade ICR mice (female, 6 weeks, 20±2 g) were randomly divided into two groups (n = 15) and treated with LUN@RBP$_{P545}$ (20 mg/kg) or sterile saline twice at 0 and 14 days post primary immunization (dppi). At 28 dppi, blood was collected from the tails of mice (200 µL per mouse), and serum was separated to detect the levels of RBP$_{P545}$-specific IgG, IgM, and IgA. After that, the sterile saline and LUN@RBP$_{P545}$ treated mice were randomly divided into three groups (n = 5), respectively. Then, one group of sterile saline treated mice and one group of LUN@RBP$_{P545}$ treated mice were infected intratracheally with carbapenem-resistant *K. pneumoniae* (CRKP, ATCC 43816) at a dose of 6×10^{9} c.f.u. per mouse. At 24 h post-infection, mice were treated with LUN@RBP$_{P545}$ (4 mg per mouse) via intravenous injection. The sterile saline and LUN@RBP$_{P545}$ treated mice without CRKP infection were treated with LUN@RBP$_{P545}$ (4 mg per mouse), or 0.1 M PBS as controls. After circulation for 30 min, the mice were sacrificed, and the lungs were collected. Finally, the fluorescence images of the lungs were captured by using a FUSION FX7 EDGE Imaging System.

UPSN@CBD$_{SA97}$ reapplication potential study

Thirty healthy SPF-grade ICR mice (female, 6 weeks, 20±2 g) were randomly divided into two groups (n = 15) and treated with UPSN@CBD$_{SA97}$ (20 mg/kg) or sterile saline twice at 0 and 14 dppi. At 28 dppi, blood was collected from the tails of mice (200 µL

per mouse), and serum was separated to detect the levels of CBD_{SA97}-specific IgG, IgM, and IgA. After that, the sterile saline and UPSN@CBD_{SA97} treated mice were randomly divided into three groups (n = 5), respectively. Then, one group of sterile saline treated mice and one group of UPSN@CBD_{SA97} treated mice were infected intratracheally with methicillin-resistant *S. aureus* (MRSA, ATCC 43300) at a dose of 6×10^9 c.f.u. per mouse. At 24 h post-infection, mice were treated with UPSN@CBD_{SA97} (4 mg per mouse) via intravenous injection. The sterile saline and UPSN@CBD_{SA97} treated mice without MRSA infection were treated with UPSN@CBD_{SA97} (4 mg per mouse), or 0.1 M PBS as controls. After circulation for 30 min, the mice were sacrificed, and the lungs were collected. Last, the fluorescence images of the lungs were captured by using a FUSION FX7 EDGE Imaging System.

Enzyme linked immunosorbent assay (ELISA)

To evaluate the RBP_{P545}-specific and CBD_{SA97}-specific antibodies (IgG, IgM, and IgA), an ELISA analysis was performed. Briefly, 100 μL of $gRBP_{P545}$ or $gCBD_{SA97}$ (1 μg/mL in carbonate solution buffer) was coated overnight to 96-well polystyrene microplates at 4 °C. After washing with phosphate-buffered saline supplemented with 0.1% Tween 20 (PBST) and blocking with 3% BSA, 100 μL of 1000-fold diluted serum samples (for IgG measurements) or 100-fold diluted serum samples (for IgM and IgA measurements) were added to the wells and incubated at 37 °C for 1 h. Subsequently, 1:5000 diluted horseradish peroxidase-conjugated goat anti-mouse IgG/IgM, or 1:1500 diluted horseradish peroxidase-conjugated goat anti-mouse IgA was added to the wells and incubated at 37 °C for 1 h. After three times washing with PBST, 100 μL

TMB solution was added and incubated at 37 °C for 15 min in the dark, followed by the addition of 50 μL 2M H_2SO_4 to stop the reaction. After that, the OD_{450} was measured by a microplate reader (Thermo Scientific, USA).

Mouse pneumonia infections and treatments

Therapeutic efficacy study of Imi@LUN@RBP$_{P545}$

The in *vivo* antibacterial efficacy of imipenem-loaded LUN@RBP$_{P545}$ (Imi@LUN@RBP$_{P545}$) was assessed in a CRKP-induced mouse pneumonia model. Briefly, SPF-grade ICR mice (female, 6 weeks, 20±2 g, n=10 per group) were infected intratracheally with CRKP (ATCC 43816) at a dose (8×10^9 c.f.u. per mouse) that leads to 80% mortality 12-48h post infection. At 12 h post-infection, mice were treated with Imi@LUN@RBP$_{P545}$ (40mg/kg), Imi@LUN@RBP$_{P545}$ (20mg/kg), Imi@LUN@RBP$_{P545}$ (10mg/kg), Imi@LUN (20mg/kg), free imipenem (20mg/kg), or 0.1 M PBS via intravenous injection. Mice without CRKP infection were used as the non-infection control. The survival rates of different groups were monitored for 14 days.

To get a deeper insight into the antibacterial efficacy of Imi@LUN@RBP$_{P545}$ in *vivo*, SPF-grade ICR mice (female, 6 weeks, 20±2 g, n=6 per group) were infected intratracheally with CRKP (ATCC 43816) at a dose (8×10^9 c.f.u. per mouse). At 12 h post-infection, mice were treated with Imi@LUN@RBP$_{P545}$ (20mg/kg), Imi@LUN (20mg/kg), free imipenem (20mg/kg), or 0.1 M PBS via intravenous injection. Mice without CRKP infection were used as the non-infection control. At 24h post-infection, organs, including heart, liver, spleen, lung, and kidney, were collected to measure the bacterial load.

Therapeutic efficacy study of Amp@UPSN@CBD$_{SA97}$

The in *vivo* antibacterial efficacy of ampicillin-loaded UPSN@CBD$_{SA97}$ (Amp@UPSN@CBD$_{SA97}$) was assessed in a MRSA-induced mouse pneumonia model. Briefly, SPF-grade ICR mice (female, 6 weeks, 20±2 g, n=10 per group) were infected intratracheally with MRSA (ATCC 43300) at a dose (1×10^{10} c.f.u. per mouse) that leads to 80% mortality 12-48h post infection. At 12 h post-infection, mice were treated with Amp@UPSN@CBD$_{SA97}$ (40mg/kg), Amp@UPSN@CBD$_{SA97}$ (20mg/kg), Amp@UPSN@CBD$_{SA97}$ (10mg/kg), Amp@UPSN (20mg/kg), free ampicillin (20mg/kg), or 0.1 M PBS via intravenous injection. Mice without MRSA infection were used as the non-infection control. The survival rates of different groups were monitored for 14 days.

To get a deeper insight into the antibacterial efficacy of Amp@UPSN@CBD$_{SA97}$ in *vivo*, SPF-grade ICR mice (female, 6 weeks, 20±2 g, n=6 per group) were infected intratracheally with MRSA (ATCC 43300) at a dose (1×10^{10} c.f.u. per mouse) that leads to 80% mortality 12-48h post infection. At 12 h post-infection, mice were treated with Amp@UPSN@CBD$_{SA97}$ (20mg/kg), Amp@UPSN (20mg/kg), free ampicillin (20mg/kg), or 0.1 M PBS via intravenous injection. Mice without MRSA infection were used as the non-infection control. At 24h post-infection, organs, including heart, liver, spleen, lung, and kidney, were collected to measure the bacterial load.

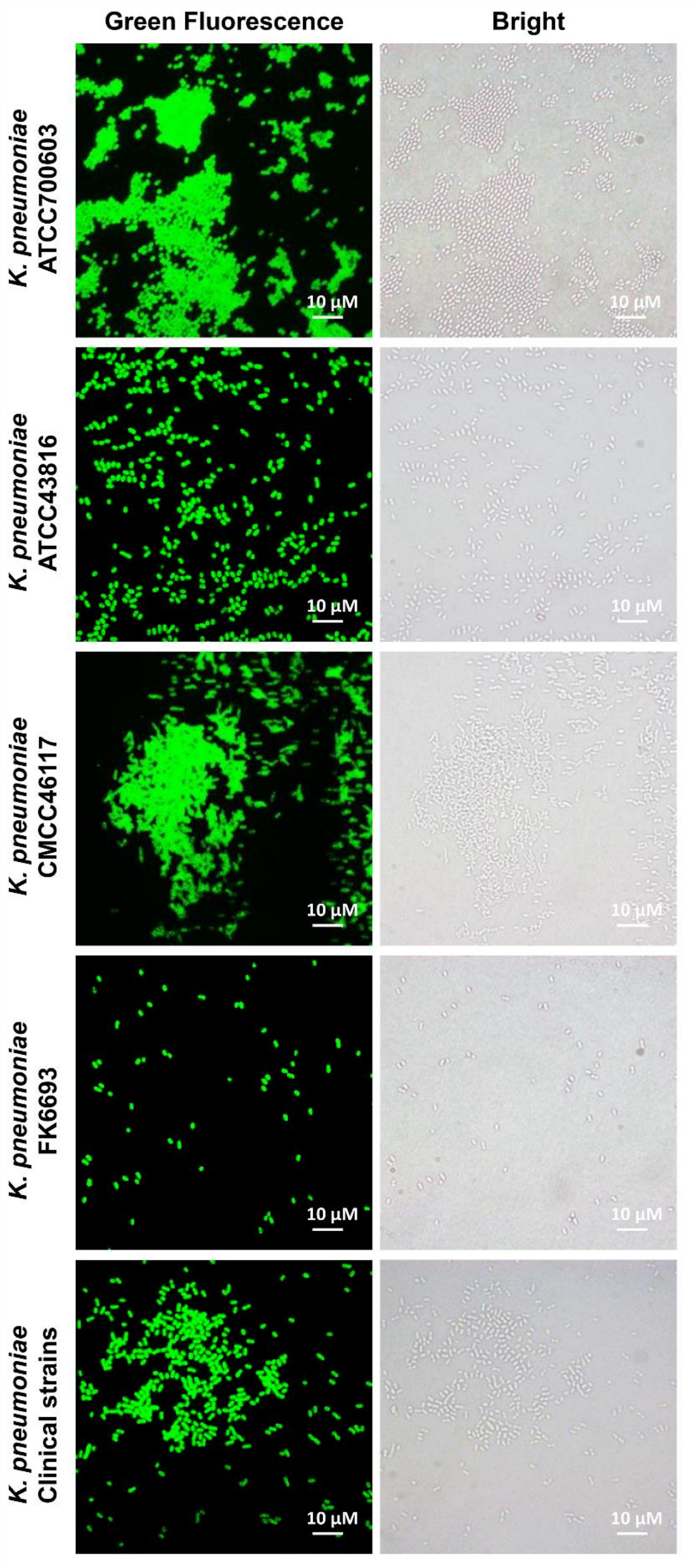

Supplementary Fig. 1: Fluorescence microscopy images of *K. pneumoniae* cells after treatment of $gRBP_{P545}$ at 37 °C for 30 min. The observed green cells demonstrate that $gRBP_{P545}$ was bonded to all five tested *K. pneumoniae* strains. (Scale bars represent 10 μm). Three times the experiment was repeated with similar results.

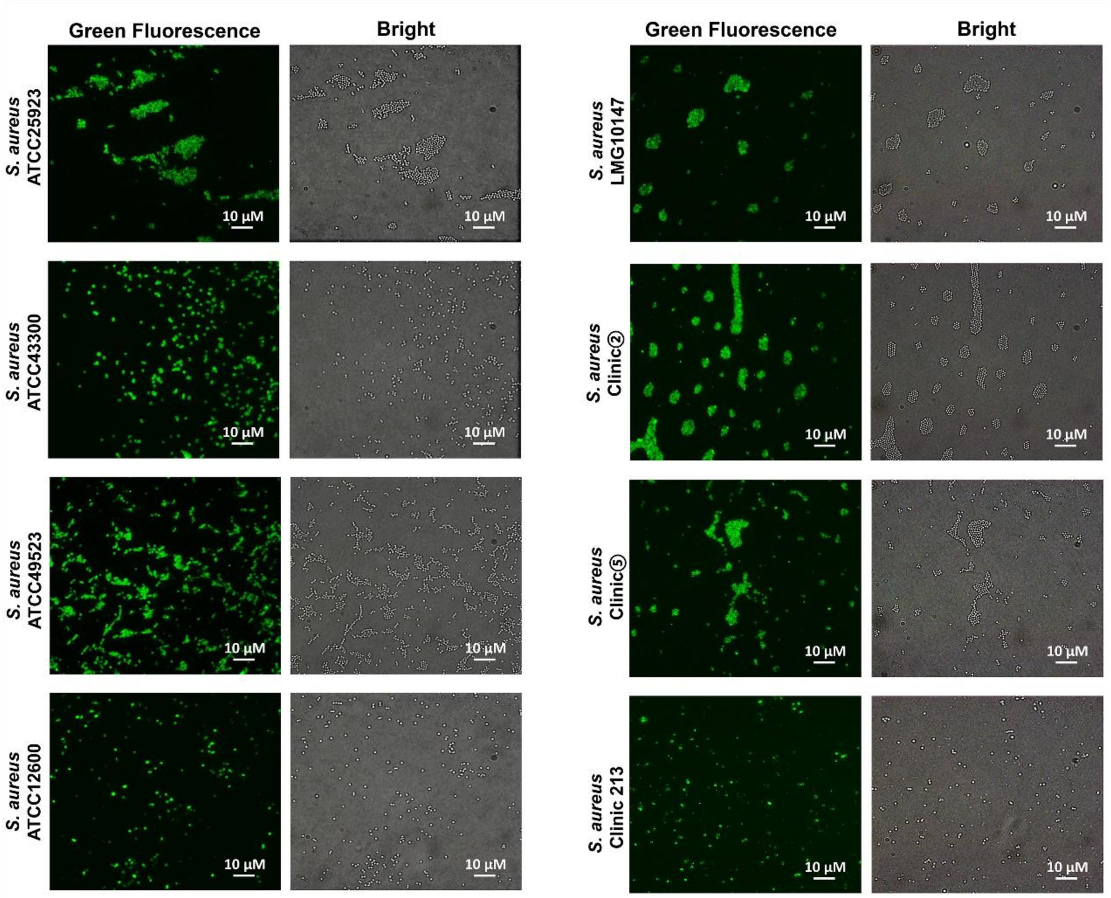

Supplementary Fig. 2: Fluorescence microscopy images of *S. aureus* cells after treatment of $gCBD_{SA97}$ at 37 °C for 30 min. The observed green cells demonstrate that $gCBD_{SA97}$ was bonded to all eight tested *S. aureus* strains. (Scale bars represent 10 μm). Three times the experiment was repeated with similar results.

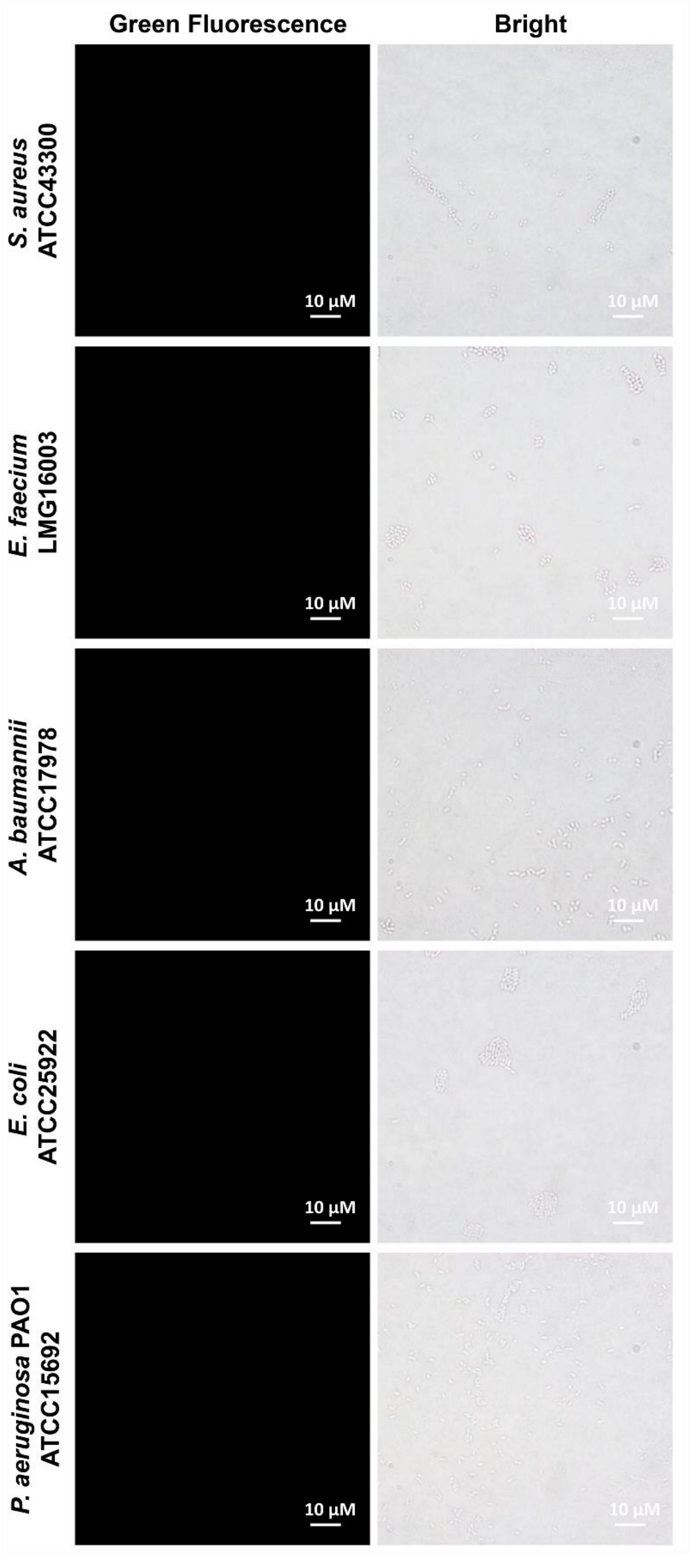

Supplementary Fig. 3: Fluorescence microscopy images of *S. aureus*, *E. faecium*, *A. baumannii*, *E. coli*, *P. aeruginosa* PAO1 after treatment of gRBP$_{P545}$ at 37 °C for 30 min. No green cells were observed, demonstrating gRBP$_{P545}$ was specifically bonded to *K. pneumoniae* strains. (Scale bars represent 10 μm). Three times the experiment was repeated with similar results.

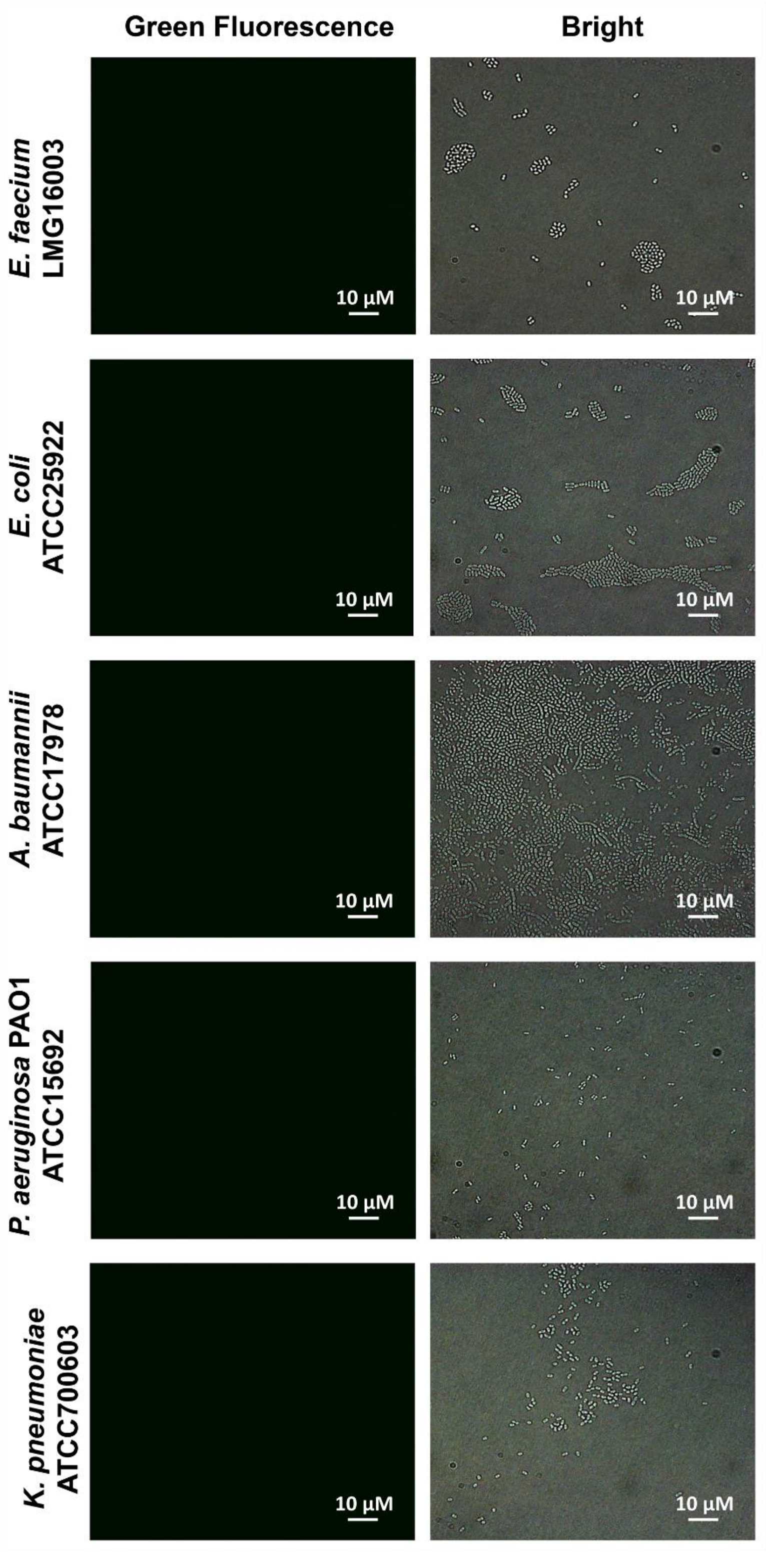

Supplementary Fig. 4: Fluorescence microscopy images of *E. faecium*, *K. pneumoniae*, *A. baumannii*, *E. coli*, *P. aeruginosa* PAO1 after treatment of $gCBD_{SA97}$ at 37 °C for 30 min. No green cells were observed, demonstrating $gCBD_{SA97}$ was specifically bonded to *S. aureus* strains. (Scale bars represent 10 μm). Three times the experiment was repeated with similar results.

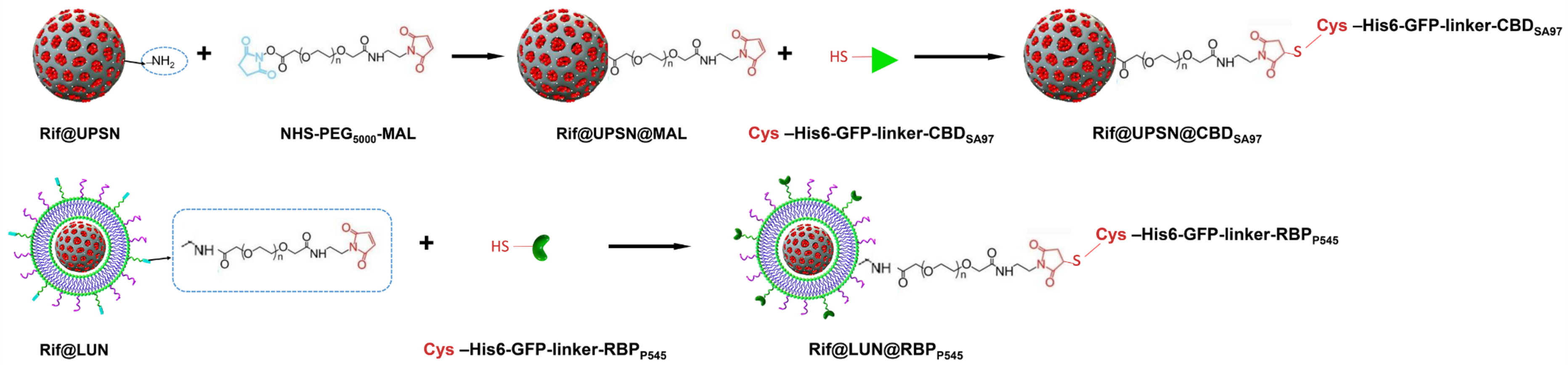

Supplementary Fig. 5: Schematic illustration depicting gCBD$_{SA97}$ (Cys-His6-GFP-linker-CBD$_{SA97}$) conjugates to Rif@UPSN and gRBP$_{P545}$ (Cys-His6-GFP-linker-RBP$_{P545}$) conjugates to Rif@LUN. gCBD$_{SA97}$ conjugates to maleimide-activated Rif@UPSN via the designed free cysteine residue at the N-terminus of this protein, and gRBP$_{P545}$ conjugates to maleimide-activated Rif@LUN via the designed free cysteine residue at the N-terminus of this protein.

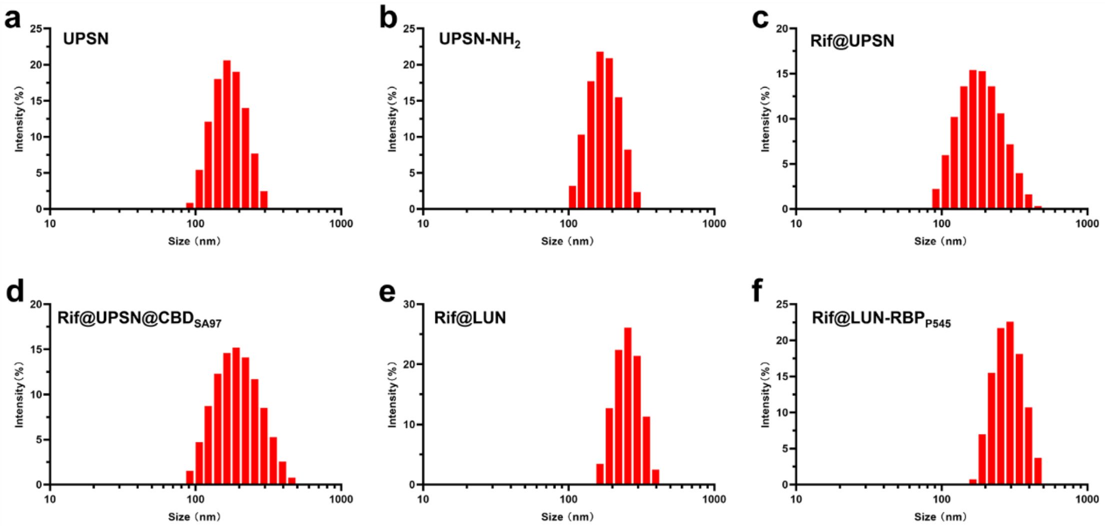

Supplementary Fig. 6: Hydrodynamic size distribution of the nanoparticles measured by dynamic light scattering. **a**, The hydrodynamic diameters of UPSN. **b**, The hydrodynamic diameters of UPSN-NH_2. **c**, The hydrodynamic diameters of Rif@UPSN. **d**, The hydrodynamic diameters of Rif@UPSN@CBD$_{SA97}$. **e**, The hydrodynamic diameters of Rif@LUN. **f**, The hydrodynamic diameters of Rif@LUN@RBP$_{P545}$. Source data are provided as a Source Data file.

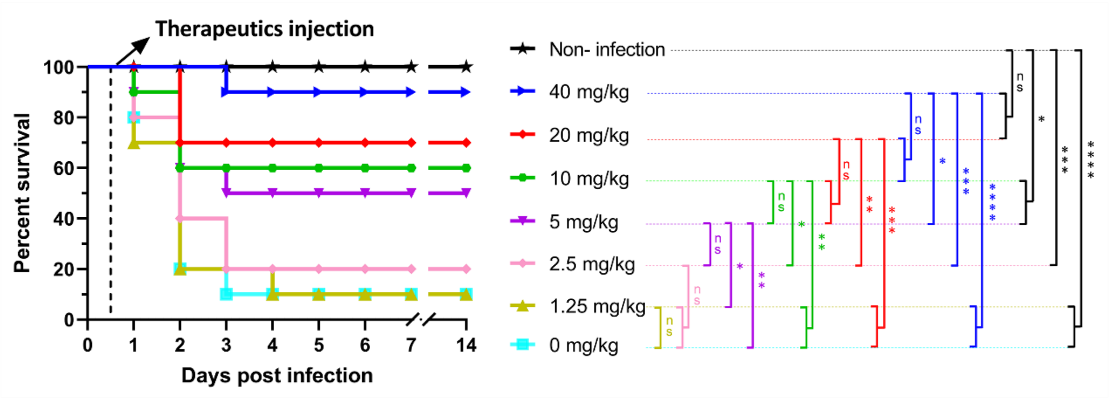

Supplementary Fig. 7: Survival rate (n = 10 biological replicates) of mice after intratracheal inoculation of CRKP at a dose of 8×10^9 c.f.u. per mouse. Groups of mice received free rifampicin at a dose range of 1.25 to 40 mg/kg intravenously 12 h post the bacterial inoculation. Survival was analyzed by the Log-rank (Mantel-Cox) test. ns, no significance; *, $p < 0.05$;**, $p < 0.01$; ***, $p < 0.001$; ****, $p < 0.0001$. Source data are provided as a Source Data file.

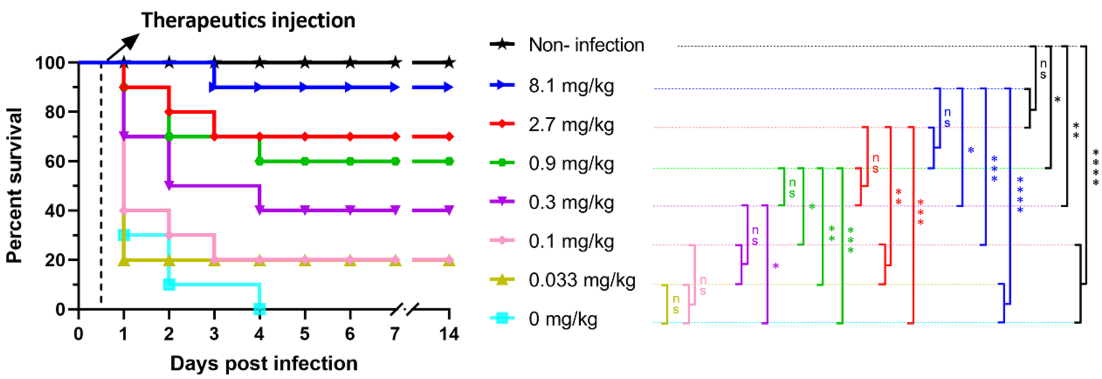

Supplementary Fig. 8: Survival rate (n = 10 biological replicates) of mice after intratracheal inoculation of MRSA at a dose of 1×10^{10} c.f.u. per mouse. Groups of mice received free rifampicin at a dose range of 0.033 to 8.1 mg/kg intravenously 12 h post the bacterial inoculation. Survival was analyzed by the Log-rank (Mantel-Cox) test. ns, no significance; *, p < 0.05;**, p < 0.01; ***, p < 0.001; ****, p < 0.0001. Source data are provided as a Source Data file.

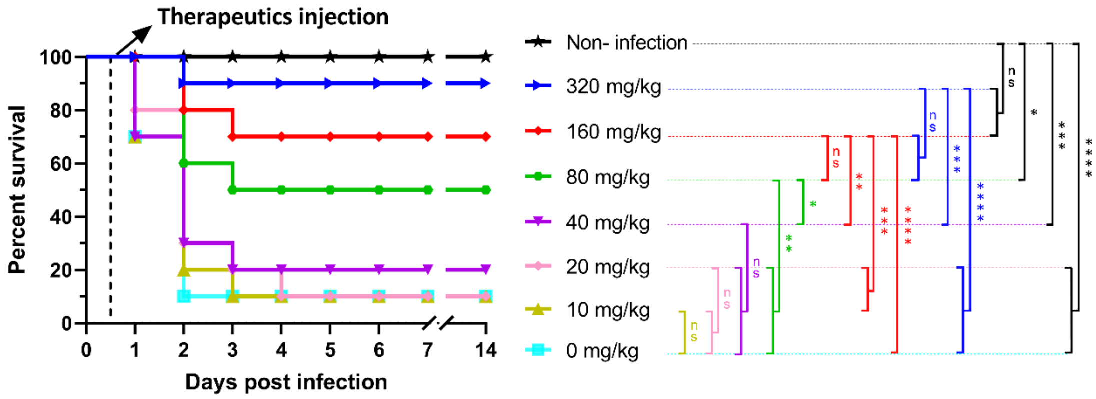

Supplementary Fig. 9: Survival rate (n = 10 biological replicates) of mice after intratracheal inoculation of CRKP at a dose of 8×10^9 c.f.u. per mouse. Groups of mice received free imipenem at a dose range of 10 to 320 mg/kg intravenously 12 h post the bacterial inoculation. Survival was analyzed by the Log-rank (Mantel-Cox) test. ns, no significance; *, p < 0.05;**, p < 0.01; ***, p < 0.001; ****, p < 0.0001. Source data are provided as a Source Data file.

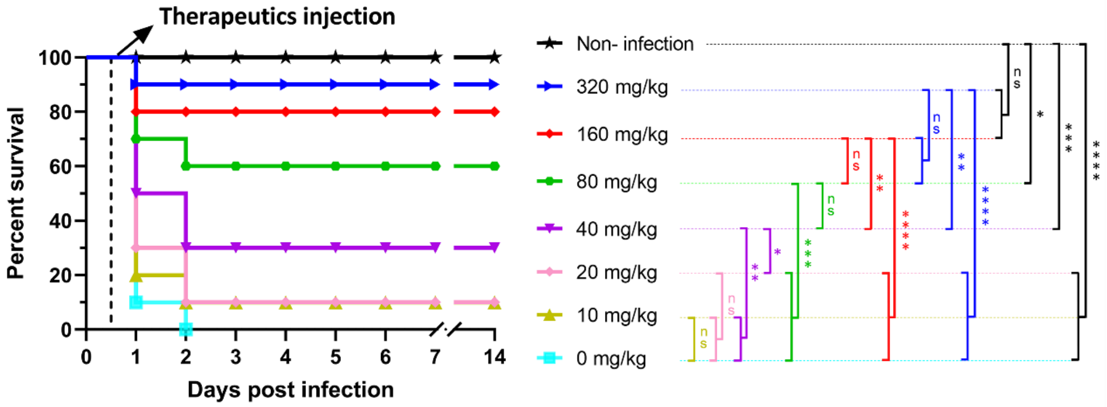

Supplementary Fig. 10: Survival rate (n = 10 biological replicates) of mice after intratracheal inoculation of MRSA at a dose of 1×10^{10} c.f.u. per mouse. Groups of mice received free ampicillin at a dose range of 10 to 320 mg/kg intravenously 12 h post the bacterial inoculation. Survival was analyzed by the Log-rank (Mantel-Cox) test. ns, no significance; *, $p < 0.05$;**, $p < 0.01$; ***, $p < 0.001$; ****, $p < 0.0001$. Source data are provided as a Source Data file.

Supplementary Table 1. Gene sequences of RBP_{P545} and CBD_{SA97}.

Gene	Sequence
RBP_{P545}	ATGGCTCAAAATAACTTTCAGCACTATAGTGACTTAGCTGAATACACAATTTTTAAACCG<br>GCTAATACAGATTGGCCGGATTCTGTGGATAATGTCCAAAAAGCATTATCAATGCTTGGC<br>GACTGGTCACGTACTGACGTAGGTTTACCTATAGCATCTCCTACAACTCCGGGTATTGTG<br>GCTATAGCTACTCAACCTGAAGTAGATGCAGGGGTAATAACAAATAAATTTGTTAGCCCA<br>GCCACATTAAAATCTGTAATCACTAGGCCAGATGCAACTACCACAGTAAAAGGCCTAAC<br>CAGATATGCTACTAATGCTGAAGCAGCCGGATTCGCTATTGGCAACGCCGCTATAGTTCC<br>TTCTGCATTAGGCCATGTTTTTACCAATGTAACAGCAAATGAAACTAGATTTGGTACTATT<br>AAGCGTGCCACTTATGATATGGGACAAGCTGGTACTGATGATACTACAGCAGTAACTCCT<br>AAACGCGTTGTAGAAATGATTGCCGCTCATTCAGCTAATCCTCCTTCATATACGATGGCG<br>ACAGAAACTAACTTAGGGTTGGTTAAGTTAGCTACTAGCGCTCAAATTCAGCAAGGGA<br>CTTTAGGAGATGGATACGCTATATCACCTAAAGGATTACTTGGTGCTAAAGCATCTGAAT<br>CTAAATTTGGCGTTATTCGTTTATCTACTGCATCTGAAACAGCTGCCGGGACTTTAGATTC<br>TGTAGCAGTTTCTCCTAAGCAATTGATGGCCCTTCGCGGTTCTACATCCCAATATGGGTT<br>AGTCCAGCTATCTGGTGTTCCTCAATCAGGCCAAACTGCCGCAAGAGCTGATGCCGTCG<br>TGTTTAAAACAACTACCGTAAACGGTAAGCCTTTATCTGGAAATATTACTTTAGGTGCTG<br>GGGATGTAAACGCTTGGTCTAAAGGCGAAGCTGACAACCGTTTTATGCCTAAAGACAG<br>GATGGTTGGCAATATTACCCGCATTGAAGGAGCTACCTACCTTAACTACAGAGGAAACA<br>ACGGTAGTCCAGATACTTGGTATTGCCAAAGTCCGTGGGAAGGCTCTTCTACTGTAGGT<br>ATGAACGTTGTTTGTAAATTTGAAAGAAACAATGATGGCGGCGATAACCGTATTTTTCA<br>GTTCTATGTTCGTGTTAATGGGCAAAGACAAGGCGGAATGCTTACATTAAATATAGAAA<br>ACACTAAAGGCGGAAGAAATGGTCATTCCTGGAGATTTGAGGCTTATGCGTCGGGCAA<br>TTTCCGGTTTGGTAACATTCCTCCAGGAGCTAGAGTTGACATTGAGCCTGTCCAATGGC<br>ACCGAGTTTTACATGTAGACGTACTGGCCACATTCTGCACAAAT
CBD_{SA97}	AAGCCAAGCGCTGACAAAATAACATGGAATTGGAAAGGCGTATTTTATCCTAATCCAGA<br>AAAAGCTATAAGAGTCAGAAAAACAGCTGGATTAACCGGCACAGTCGTTGAAGAAGAT<br>TCATGGCTATACACAAAAGATGATTGGGTAAAATTCGACCAAGTCATTAAAAAAGATGG<br>CTACTGGTGGATTAGATTCAAATATCAACGTGAGGGCTCTAGTACTAACAATTTCTATTGT<br>GCAGTGTGTAGAATTACTGATAAGGAACAAAAGATTAAAAATGAAAAATATTGGGGCAC<br>GATTGAGTGGGCT

Supplementary Table 2. Amino acid sequence of $gRBP_{P545}$ and $gCBD_{SA97}$.

protein	Amino Acid Sequence
$gRBP_{P545}$	MCGSSHHHHHHSQDPMSKGEELFTGVVPILVELDGDVNGHKFSVRGEGEGDATNGKLTLK<br>FICTTGKLPVPWPTLVTTLTYGVQCFSRYPDHMKRHDFFKSAMPEGYVQERTISFKDDGTYK<br>TRAEVKFEGDTLVNRIELKGIDFKEDGNILGHKLEYNFNSHNVYITADKQKNGIKANFKIRHN<br>VEDGSVQLADHYQQNTPIGDGPVLLPDNHYLSTQSVLSKDPNEKRDHMVLLEFVTAAGITH<br>GMDELYKGSGGGGS**MAQNNFQHYSDLAEYTIFKPANTDWPDSVDNVQKALSMLGDWS**<br>**RTDVGLPIASPTTPGIVAIATQPEVDAGVITNKFVSPATLKSVITRPDATTTVKGLTRYATNAE**<br>**AAGFAIGNAAIVPSALGHVFTNVTANETRFGTIKRATYDMGQAGTDDTTAVTPKRVVEMI**<br>**AAHSANPPSYTMATETNLGLVKLATSAQIQQGTLGDGYAISPKGLLGAKASESKFGVIRLST**<br>**ASETAAGTLDSVAVSPKQLMALRGSTSQYGLVQLSGVPQSGQTAARADAVVFKTTTVNG**<br>**KPLSGNITLGAGDVNAWSKGEADNRFMPKDRMVGNITRIEGATYLNYRGNNGSPDTWY**<br>**CQSPWEGSSTVGMNVVCKFERNNDGGDNRIFQFYVRVNGQRQGGMLTLNIENTKGGR**<br>**NGHSWRFEAYASGNFRFGNIPPGARVDIEPVQWHRVLHVDVLATFCTN**
$gCBD_{SA97}$	MCGSSHHHHHHSQDPMSKGEELFTGVVPILVELDGDVNGHKFSVRGEGEGDATNGKLTLK<br>FICTTGKLPVPWPTLVTTLTYGVQCFSRYPDHMKRHDFFKSAMPEGYVQERTISFKDDGTYK<br>TRAEVKFEGDTLVNRIELKGIDFKEDGNILGHKLEYNFNSHNVYITADKQKNGIKANFKIRHN<br>VEDGSVQLADHYQQNTPIGDGPVLLPDNHYLSTQSVLSKDPNEKRDHMVLLEFVTAAGITH<br>GMDELYKGSGGGGS**KPSADKITWNWKGVFYPNPEKAIRVRKTAGLTGTVVEEDSWLYTK**<br>**DDWVKFDQVIKKDGYWWIRFKYQREGSSTNNFYCAVCRITDKEQKIKNEKYWGTIEWA**

Supplementary Table 3. Stains used in this study.

organism	Characteristics	Source
E. coli	ATCC25922	ATCC
E. coli	BL21(DE3), protein expression.	Thermo Fisher Scientific
E. coli	TOP10, plasmid construction, plasmid maintenance.	Thermo Fisher Scientific
K. pneumoniae	ATCC700603	ATCC
K. pneumoniae	ATCC43816, carbapenem-resistant.	ATCC
K. pneumoniae	CMCC46117	CMCC
K. pneumoniae	FK6693, carbapenem-resistant.	Lab collection
K. pneumoniae	Clinical isolation, carbapenem-resistant.	Lab collection
S. aureus	ATCC25923	ATCC
S. aureus	ATCC43300, methicillin-resistant, resistant to ampicillin.	ATCC
S. aureus	ATCC49523	ATCC
S. aureus	ATCC12600	ATCC
S. aureus	LMG10147	BCCM
S. aureus	Clinic②	Lab collection
S. aureus	Clinic⑤	Lab collection
S. aureus	Clinic 213, methicillin-resistant.	Lab collection
E. faecium	LMG16003	BCCM
A. baumannii	ATCC17978	ATCC
P. aeruginosa PAO1	ATCC15692	ATCC

Supplementary Table 4. Particle sizes and zeta-potentials of the synthesized nanoparticles.

Nanoparticle	Z-average (d.nm)	Zeta-Potential (mV)
UPSN	161.0±5.2	-23.0±1.5
UPSN-NH_2	168.2±1.2	8.1±0.5
Rif@UPSN	179.4±3.5	3.8±0.7
Rif@LUN	256.4±9.1	-11.8±0.5
Rif@LUN@RBP_{P545}	297.5±2.5	-11.9±0.4
Rif@UPSN@CBD_{SA97}	183.3±1.5	-5.2±0.7

The data represent two independent experiments. Source data are provided as a Source Data file.

Supplementary Table 5. Antibiotic loading efficiency by wt.%.

Nanoparticle	Rifampicin loading efficiency (wt.%)
Rif@UPSN	66.43±2.61
Rif@LUN	63.73±3.51
Rif@LUN@RBP_{P545}	63.11±2.44
Rif@UPSN@CBD_{SA97}	67.99±3.14

The data represent two independent experiments. Source data are provided as a Source Data file.

Supplementary Table 6. Protein contents of the engineered two distinct nanodelivery systems.

Nanodelivery systems	Protein content (nmol)
LUN@RBP_{P545}	2.1±0.1
UPSN@CBD_{SA97}	2.7±0.2

The data represent two independent experiments. Source data are provided as a Source Data file.

Supplementary Table 7. Plasmids used in this study.

Plasmids	Characteristics and purpose	Source
pRSF-His6-GFP	Contains a *His6-tagged GFP* gene, template for plasmid construction.	Lab collection
pRSF-His6-GFP-RBP$_{p545}$	*pRSF-His6-GFP* derivative, containing *His6-GFP-RBP$_{p545}$* gene.	This work
pRSF-His6-GFP-CBD$_{SA97}$	*pRSF-His6-GFP* derivative, containing *His6-GFP-CBD$_{SA97}$* gene.	This work
pRSF-Cys-His6-GFP-RBP$_{p545}$	*pRSF-His6-GFP-RBP$_{p545}$* derivative, has a Cys at the N-terminus of the fused protein, expression Cys-His6-GFP-RBP$_{p545}$ (gRBP$_{p545}$).	This work
pRSF-Cys-His6-GFP-CBD$_{SA97}$	*pRSF- His6-GFP-CBD$_{SA97}$* derivative, has a Cys at the N-terminus of the fused protein, expression Cys-His6-GFP-CBD$_{SA97}$ (gCBD$_{SA97}$).	This work

Supplementary Table 8. Primers used in this study.

Primers	Nucleic acid sequences (5' to 3')	Purpose
His6-GFP-rv	CGAACCACCTCCTCCACTACCCTTATAAAGCTCATCCATGCCGTG	Amplification of *pRSF-His6-GFP* backbond for inserting of *CBD*$_{SA97}$ and *RBP*$_{p545}$ genes, respectively.
His6-GFP-fw	TAATTAACCTAGGCTGCTGCCACC	
SA97_CBD-rv	CAGCCTAGGTTAATTAAGCCCACTCAATCGTGCC	Amplification of *CBD*$_{SA97}$ gene, to generate *pRSF-His6-GFP-CBD*$_{SA97}$.
SA97_CBD-fw	GTGGAGGAGGTGGTTCGAAGCCAAGCGCTGACAAAATAAC	
P545_RBP-rv	CAGCCTAGGTTAATTAATTTGTGCAGAATGTGGCCAGTAC	Amplification of *RBP*$_{P545}$ gene, to generate *pRSF-His6-GFP-RBP*$_{p545}$.
P545_RBP-fw	GTGGAGGAGGTGGTTCGATGGCTCAAAATAACTTTCAGCACTATAGTG	
Insert Cys-rv	ACACATGGTATATCTCCTTATTAAAGTTAAACAAAATTATTTCTACAGG	Amplification of *pRSF-His6-GFP-RBP*$_{p545}$ and *pRSF-His6-GFP-CBD*$_{SA97}$ for inserting of a cystine residue gene, generating of *pRSF-Cys-his6-GFP-RBP*$_{p545}$ and *pRSF-Cys-his6-GFP-CBD*$_{SA97}$.
Insert Cys-fw	AGATATACCATGTGTGGCAGCAGCCATCACCATCATC	
PRSF-T72-Seq-rv	GGTCGTTAAATAGCCGCTTATGTC	Sequencing
pRSF-T71_Seq-fw	TCACCACCCTGAATTGACTC	

References

1. Zhang, H., Ru, J., Tang, X. & Guo, H. Urchin-like Porous Silica Nanoparticles for Simultaneous Intracellular Delivery of Doxorubicin and RNase A. *ACS Appl. Nano Mater.* **3**, 9993–10002 (2020).
2. Chen, H. *et al.* Cartilage matrix-inspired biomimetic superlubricated nanospheres for treatment of osteoarthritis. *Biomaterials* **242**, 119931 (2020).
3. Ling, L. L. *et al.* A new antibiotic kills pathogens without detectable resistance. *Nature* **517**, 455–459 (2015).
