# Supplementary material for: Guiding antibiotics towards their target using bacteriophage proteins
Source: Nat Commun. 2024 Jun 20;15:5287. doi: 10.1038/s41467-024-49603-4 (PMC11190222; doi:10.1038/s41467-024-49603-4)
Supplement: Supplementary file 3 — Reporting Summary [file 41467_2024_49603_MOESM3_ESM.pdf]

nature portfolio

Corresponding author(s): Xinghong Zhao, Zhongqiong Yin, Yong Liu, Hongping Wan

Last updated by author(s): May 28, 2024

Reporting Summary

Nature Portfolio wishes to improve the reproducibility of the work that we publish. This form provides structure for consistency and transparency in reporting. For further information on Nature Portfolio policies, see our Editorial Policies and the Editorial Policy Checklist.

Statistics

For all statistical analyses, confirm that the following items are present in the figure legend, table legend, main text, or Methods section.

n/a	Confirmed	
☐	☒	The exact sample size (*n*) for each experimental group/condition, given as a discrete number and unit of measurement
☒	☐	A statement on whether measurements were taken from distinct samples or whether the same sample was measured repeatedly
☐	☒	The statistical test(s) used AND whether they are one- or two-sided<br>*Only common tests should be described solely by name; describe more complex techniques in the Methods section.*
☒	☐	A description of all covariates tested
☒	☐	A description of any assumptions or corrections, such as tests of normality and adjustment for multiple comparisons
☐	☒	A full description of the statistical parameters including central tendency (e.g. means) or other basic estimates (e.g. regression coefficient) AND variation (e.g. standard deviation) or associated estimates of uncertainty (e.g. confidence intervals)
☐	☒	For null hypothesis testing, the test statistic (e.g. *F*, *t*, *r*) with confidence intervals, effect sizes, degrees of freedom and *P* value noted<br>*Give P values as exact values whenever suitable.*
☒	☐	For Bayesian analysis, information on the choice of priors and Markov chain Monte Carlo settings
☒	☐	For hierarchical and complex designs, identification of the appropriate level for tests and full reporting of outcomes
☐	☒	Estimates of effect sizes (e.g. Cohen's *d*, Pearson's *r*), indicating how they were calculated

Our web collection on statistics for biologists contains articles on many of the points above.

Software and code

Policy information about availability of computer code

Data collection	The OD450nm, OD562nm, and OD570nm values were measured using a Thermo Scientific Varioskan Flash multimode microplate reader; the concentration of antibiotic solution samples was measured using an Agilent 1260 Infinity high performance liquid chromatography system; fluorescent images of bacteria were obtained by STELLARIS STED/EM CPD300 confocal microscope system (Leica, Germany) or Nikon 80i (Japan) microscope system; the fluorescence images of the lungs were captured by using a FUSION FX7 EDGE Imaging System; a transmission electron microscopy instrument system (JEM-2100plus, Japan) operating at 120kV accelerating voltage was used to record TEM images; the hydrodynamic diameters and zeta potentials of nanoparticles were measured using a Malvern Zetasizer Nano ZSE system (Malvern Instruments); the haematoxylin and eosin staining of histology sections from major organs were captured using a Olympus VS120 Virtual Slide Microscope system; the western results were collected using a ChemiDoc MP Imaging System (Bio-Rad).
Data analysis	GraphPad Prism 8 was used to plot graphs. All the statistical analyses were performed using GraphPad Prism 8 software (GraphPad Software). All data represent mean value ± standard deviation. The statistical significance of the data was assessed using one-way ANOVA followed by Tukey's multiple comparisons test with GraphPad Prism 8.0. Survival was analyzed by the Log-rank (Mantel-Cox) test with GraphPad Prism 8.0. ns, no significance; *, p<0.05; **, p<0.01; ***, p<0.001; ****, p<0.0001.

For manuscripts utilizing custom algorithms or software that are central to the research but not yet described in published literature, software must be made available to editors and reviewers. We strongly encourage code deposition in a community repository (e.g. GitHub). See the Nature Portfolio guidelines for submitting code & software for further information.

Data

Policy information about availability of data

All manuscripts must include a data availability statement. This statement should provide the following information, where applicable:

- Accession codes, unique identifiers, or web links for publicly available datasets
- A description of any restrictions on data availability
- For clinical datasets or third party data, please ensure that the statement adheres to our policy

The authors declare that all data supporting the findings of this study are available within the paper and its Supplementary Information.

Research involving human participants, their data, or biological material

Policy information about studies with human participants or human data. See also policy information about sex, gender (identity/presentation), and sexual orientation and race, ethnicity and racism.

Reporting on sex and gender	n/a
Reporting on race, ethnicity, or other socially relevant groupings	n/a
Population characteristics	n/a
Recruitment	n/a
Ethics oversight	n/a

Note that full information on the approval of the study protocol must also be provided in the manuscript.

Field-specific reporting

Please select the one below that is the best fit for your research. If you are not sure, read the appropriate sections before making your selection.

☒ Life sciences ☐ Behavioural & social sciences ☐ Ecological, evolutionary & environmental sciences

For a reference copy of the document with all sections, see nature.com/documents/nr-reporting-summary-flat.pdf

Life sciences study design

All studies must disclose on these points even when the disclosure is negative.

Sample size	No statistical methods was used to predetermine the samples size. The sample sizes were determined as minimal to lower the cost and be sufficient to obtain statistically significant difference between experimental groups (n=3-10). For property measurement experiments, samples were prepared and tested at least twice. For in vivo studies, each group contains at least 3 for evaluating the statistical significance. These sample sizes also represent the standard practice for publication in this field and were described in figure legends. Each sample represents independent biological replicates.
Data exclusions	No data were excluded from the analyses.
Replication	All experiments were repeated at least three times and all attempts at replication generated similar results.
Randomization	The samples were randomly grouped.
Blinding	No blinding was required because all measurements are not subject to investigator's bias or ambiguity.

Reporting for specific materials, systems and methods

We require information from authors about some types of materials, experimental systems and methods used in many studies. Here, indicate whether each material, system or method listed is relevant to your study. If you are not sure if a list item applies to your research, read the appropriate section before selecting a response.

Materials & experimental systems

n/a	Involved in the study
☐	☒ Antibodies
☐	☒ Eukaryotic cell lines
☒	☐ Palaeontology and archaeology
☐	☒ Animals and other organisms
☒	☐ Clinical data
☒	☐ Dual use research of concern
☒	☐ Plants

Methods

n/a	Involved in the study
☒	☐ ChIP-seq
☒	☐ Flow cytometry
☒	☐ MRI-based neuroimaging

Antibodies

Antibodies used	His-Tag (6*His) Monoclonal antibody (Cat No. 66005-1-lg), horseradish peroxidase-conjugated Goat Anti-Mouse IgG (Cat No. SA00001-1), horseradish peroxidase-conjugated Goat Anti-Mouse IgM (Cat No. SA00012-6), and horseradish peroxidase-conjugated Goat Anti-Mouse IgA (Cat No. SA00012-7) were purchased from Proteintech Group, Inc (Rosemont, USA).
Validation	His-Tag (6*His) Monoclonal antibody (Cat No. 66005-1-lg, https://www.ptgcn.com/products/His-Tag-Antibody-66005-1-Ig.htm): Positive WB detected in recombinant protein; Application, Western Blot (WB); Suggested Dilution Range, WB 1:5000-1:50000. Horseradish peroxidase-conjugated Goat Anti-Mouse IgG (Cat No. SA00001-1, https://www.ptgcn.com/products/HRP-conjugated-Affinipure-Goat-Anti-Mouse-IgG-H-L-secondary-antibody.htm): Species Reactivity, Mouse; Suggested Dilution Range, 1:1000-1:20,000 for ELISA and Western blotting with chromogenic substrates. Horseradish peroxidase-conjugated Goat Anti-Mouse IgM (Cat No. SA00012-6, https://www.ptgcn.com/products/Peroxidase-conjugated-Affinipure-Goat-Anti-Mouse-IgM-Chain-Specific.htm): Species Reactivity, Mouse; Suggested Dilution Range, 1:1000-1:20,000 for ELISA with chromogenic substrates. Horseradish peroxidase-conjugated Goat Anti-Mouse IgA (Cat No. SA00012-7, https://www.ptgcn.com/products/Peroxidase-conjugated-Affinipure-Goat-Anti-Mouse-IgA-.htm): Species Reactivity, Mouse; Suggested Dilution Range, 1:250-1:1500 for ELISA for ELISA with chromogenic substrates.

Eukaryotic cell lines

Policy information about cell lines and Sex and Gender in Research

Cell line source(s)	Hepatoblastoma cell line (Hep G2, ATCC HB-8065) and human embryonic kidney 293T (HEK-293T, ATCC CRL-3216) were obtained from the American Type Culture Collection (ATCC).
Authentication	None of the cell lines were authenticated.
Mycoplasma contamination	The cell lines were not tested for mycoplasma contamination.
Commonly misidentified lines (See ICLAC register)	No commonly misidentified species used.

Animals and other research organisms

Policy information about studies involving animals; ARRIVE guidelines recommended for reporting animal research, and Sex and Gender in Research

Laboratory animals	Six-week old mice (SPF-grade ICR, female) were purchased from Chengdu Dossy Experimental Animals Co., Ltd. All mice were housed in the Animal Center of the College of Veterinary Medicine, Sichuan Agricultural University under standard conditions with free access to food and water. The light was from 8:00 am to 8:00 pm, with the temperature kept at 22±1°C and humidity at 40–70%. All experimental procedures involving animals were in accordance with the guidelines of the Animal Care and Use Committee of Sichuan Agricultural University. All animal experiments were performed independently of each other with different cohorts of mice.
Wild animals	None.
Reporting on sex	SPF-grade ICR mice (female, 6 weeks, 20±2 g).
Field-collected samples	None.
Ethics oversight	All animal experiments conformed to the Guide for the Care and Use of Laboratory Animals from the National Institutes of Health, and all procedures were approved by the Animal Research Committee of Sichuan Agricultural University [permission number 20230074]. The use of New Zealand white rabbit biological materials (erythrocytes isolated from the blood of healthy rabbits) for research was approved by Sichuan Agricultural University Institution Review Board.

Note that full information on the approval of the study protocol must also be provided in the manuscript.

Plants

Seed stocks	n/a
Novel plant genotypes	n/a
Authentication	n/a
